# Regulation of actin cytoskeleton architecture by Eps8 and Abi1

**DOI:** 10.1186/1471-2121-6-36

**Published:** 2005-10-14

**Authors:** Julaine Roffers-Agarwal, Jennifer B Xanthos, Jeffrey R Miller

**Affiliations:** 1 Department of Genetics, Cell Biology, and Development, University of Minnesota, 6-160 Jackson Hall, 321 Church St SE, Minneapolis, MN 55455, USA

## Abstract

**Background:**

The actin cytoskeleton participates in many fundamental processes including the regulation of cell shape, motility, and adhesion. The remodeling of the actin cytoskeleton is dependent on actin binding proteins, which organize actin filaments into specific structures that allow them to perform various specialized functions. The Eps8 family of proteins is implicated in the regulation of actin cytoskeleton remodeling during cell migration, yet the precise mechanism by which Eps8 regulates actin organization and remodeling remains elusive.

**Results:**

Here, we show that Eps8 promotes the assembly of actin rich filopodia-like structures and actin cables in cultured mammalian cells and *Xenopus *embryos, respectively. The morphology of actin structures induced by Eps8 was modulated by interactions with Abi1, which stimulated formation of actin cables in cultured cells and star-like structures in *Xenopus*. The actin stars observed in *Xenopus *animal cap cells assembled at the apical surface of epithelial cells in a Rac-independent manner and their formation was accompanied by recruitment of N-WASP, suggesting that the Eps8/Abi1 complex is capable of regulating the localization and/or activity of actin nucleators. We also found that Eps8 recruits Dishevelled to the plasma membrane and actin filaments suggesting that Eps8 might participate in non-canonical Wnt/Polarity signaling. Consistent with this idea, mis-expression of Eps8 in dorsal regions of *Xenopus *embryos resulted in gastrulation defects.

**Conclusion:**

Together, these results suggest that Eps8 plays multiple roles in modulating actin filament organization, possibly through its interaction with distinct sets of actin regulatory complexes. Furthermore, the finding that Eps8 interacts with Dsh and induced gastrulation defects provides evidence that Eps8 might participate in non-canonical Wnt signaling to control cell movements during vertebrate development.

## Background

Remodeling of the actin cytoskeleton is critical for mediating changes in cell shape, migration, and adhesion. Actin filament architecture is regulated by a large group of actin binding proteins that modulate actin assembly, disassembly, branching, and bundling [1]. Actin organization is also regulated by growth factor signals that stimulate the activity of Rho family GTPases, which mediate actin remodeling and formation of stress fibers, filopodia, and membrane ruffles [2]. Although much has been learned about the general properties of actin binding proteins, the mechanisms by which these proteins control actin architecture in vivo are poorly understood.

Eps8 (EGF receptor pathway substrate 8) was originally identified as a substrate of the EGF receptor [3] and is the founding member of a multigene family of Eps8-like proteins named Eps8L1, Eps8L2, and Eps8L3 [4,5]. Eps8 is thought to transduce growth factor signals by acting as a scaffold protein to support the formation of multi-protein signaling complexes that promote the activation of Rho family GTPases. Consistent with this model, studies in Eps8 null fibroblasts showed that Eps8 is required for growth factor-induced Rac activation as well as Rac-dependent actin remodeling and membrane ruffling [6]. Eps8 is a critical component of a complex that contains the p85 regulatory subunit of phosphoinositide 3-kinase, Abi1, and Sos1, which acts as a guanine nucleotide exchange factor (GEF) for Rac [6,7]. Eps8 interacts directly with Abi1 through its SH3 domain, which possesses a novel peptide binding specificity [8], and this binding is thought to relieve auto-inhibition of Eps8 [9].

Eps8 also directly binds actin, suggesting that it may function by localizing Rac to sites of actin remodeling [10]. Eps8 binds actin through its C-terminal effector domain and expression of the effector region in serum-starved cells elicits Rac-dependent actin remodeling and membrane ruffling [10]. Studies using deletion mutants of Eps8 show that the C-terminal effector domain is required for localizing Eps8 to membrane ruffles and the transduction of signals to Rac [10]. A recent study revealed that C-terminal fragments of Eps8 also possess actin barbed-end capping activity in vitro and can substitute for capping protein in actin-based motility assays, suggesting a mechanism by which Eps8 might regulate actin filament dynamics in vivo [9]. Interestingly, full-length Eps8 on its own lacks capping activity in vitro, but can block actin polymerization in the presence of Abi1 [9]. The capping activity of Eps8 does not require Rac indicating that Eps8 can modulate actin dynamics through Rac-dependent and -independent mechanisms. Together, these data implicate Eps8 as a key regulator of actin filament dynamics and suggest that its activity is modulated through association with distinct sets of interacting regulatory proteins.

Eps8 has also been shown to bind Dishevelled (Dsh) [11], a key regulator of canonical and non-canonical Wnt signaling [12,13]. Dsh is required for the establishment of cell polarity and directed migration during gastrulation in vertebrates [14-16]. The mechanism by which Dsh controls cell polarity and migration is unclear, but is hypothesized to involve the modulation of actin dynamics through activation of RhoA and Rac [17,18]. The ability of Eps8 to bind both Dsh and actin and stimulate Rac activation suggests that Eps8 may play an important role in regulating Dsh function during gastrulation, but this possibility has not been investigated.

In this study, we utilized cultured mammalian cells and *Xenopus *embryos as model systems to investigate the mechanism by which Eps8 regulates actin filament architecture in vivo. Our results provide evidence that Eps8 can stimulate the assembly of distinct types of actin-based structures in cells and that the morphology of the actin structures induced by Eps8 is dependent on its interactions with Abi1. In addition, we show that Eps8 can recruit actin regulatory proteins, such as N-WASP and Dsh, to actin filaments and that mis-expression of Eps8 impairs cell movements during gastrulation in *Xenopus *embryos. Together, these data suggest that the role of Eps8 in modulating actin organization is multifaceted and is dependent on its participation in several potentially distinct multi-protein actin regulatory complexes.

## Results

### Enhanced formation of filopodia-like structures in cells expressing Eps8

**Figure 1:**
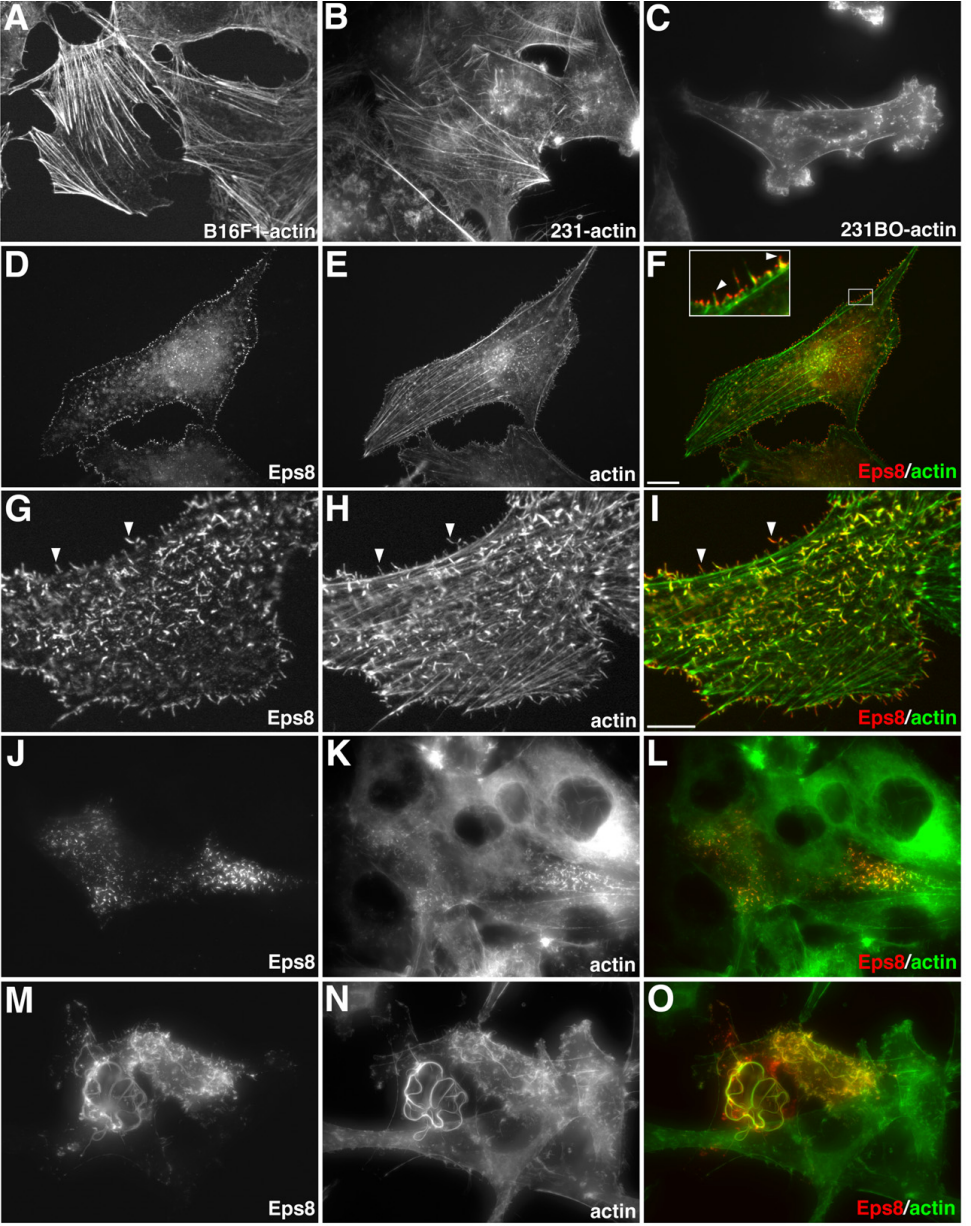
**Eps8 induced actin remodeling in cultured cells**. Phalloidin staining of the actin cytoskeleton in untransfected (A) B16F1, (B) MDA-MB231, and (C) MDA-MB231BO cells. Cells possess few filopodia-like structures extending from lateral and dorsal surfaces and do not possess cytoplasmic actin cables. Actin structures induced by Eps8 in (D-I) B16F1, (J-L) MDA-MB231, and (M-O) MDA-MB231BO cells. Distribution of Eps8-myc (D,G,J,M) revealed by 9e10 anti-*c-myc *antibody and actin (E,H,K,N) revealed by phalloidin staining in fixed cells. Right column (F,I,L,O) shows merged images with Eps8-myc in red and actin in green. The boxed region in (F) is enlarged in the inset. Eps8 induces the formation of filopodia-like structures in B16F1 and MDA-MB231 cells and localizes to filopodia-like structures, ruffles, and actin cables in MDA-MB231BO cells. In B16F1 cells, Eps8 localizes along the length of the filopodia-like structures (arrowheads in G-I) and is enriched at their tips (arrowheads, inset in F). Scale bar is equal to 10 μm in (A-F) and (J-O) and 5 μm in (G-I).

To gain insights into the role Eps8 plays in regulating actin filament architecture, we examined the effect of increasing Eps8 levels on actin remodeling in mammalian cultured cells. For these studies, we utilized the mouse melanoma cell line B16F1 [19], the human breast cancer cell line MDA-MB231 [20], and the MDA-MB231BO cell line, which is a highly metastatic, bone seeking clone of the parental line [21]. These cells were chosen because they are highly motile and express a variety of cellular protrusions including lamellipodia and filopodia. Control B16F1, MDA-MB231, and MDA-231BO cells stained for actin are shown in Figure 1. We found that expression of a *c-myc *epitope tagged version of mouse Eps8 (Eps8-myc) in B16F1 cells elicited the formation of filopodia-like structures, which stained brightly with phalloidin (Figure 1D–I). The filopodia-like structures extended from lateral and dorsal regions of the cell and Eps8 localized along the length of these protrusions and was enriched at their tips (Figure 1F, inset). Similar results were seen in MDA-MB231 (Figure 1J–L) and MDA-MB231BO (Figure 1M–O) breast cancer cells. More than 90% of the transfected cells displayed the actin phenotype shown. We also observed the formation of long, snake-like actin cables in approximately 50% of the MDA-MB231BO cells, which were typically not seen in either B16F1 cells or the parental MDA-MB231 cells.

### Abi1 modulates Eps8-dependent actin remodeling

**Figure 2:**
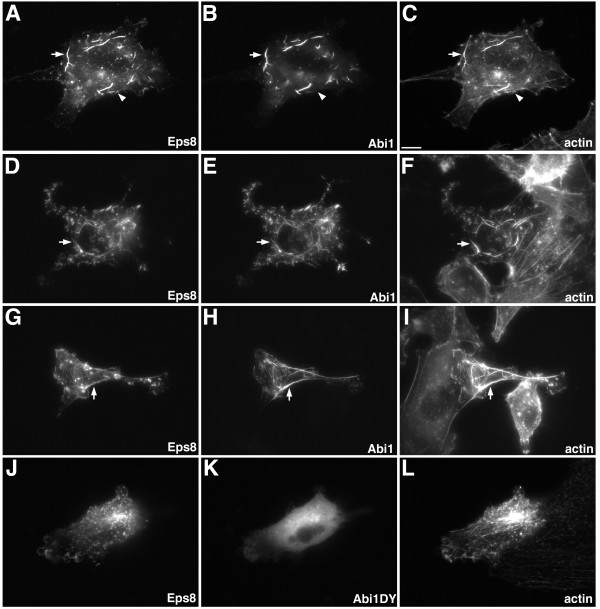
**Abi1 modulates the morphology of actin structures induced by Eps8**. Actin structures induced by Eps8 and Abi1 in (A-C) B16F1, (D-F) MDA-MB231, and (G-I) MDA-MB231BO cells. Distribution of Eps8-myc (A,D,G,J) revealed by 9e10 anti-*c-myc *antibody, Abi1-GFP (B,E,H), and Abi1DY-GFP (K) revealed by GFP, and actin (C,F,I,L) revealed by phalloidin staining in fixed cells. (A-I) Simultaneous expression of Eps8 and Abi1 induces the formation of actin cables in B16F1, MDA-MB231, and MDA-MB231BO. Eps8 and Abi1 co-localize in association with actin cables (arrows) but Abi1 is not enriched with Eps8 in filopodia (arrowheads in A-C). (J-L) Formation of actin cables in B16F1 cells is dependent on the interaction of Eps8 and Abi1. Abi1DY does not co-localize with Eps8 and does not induce actin cable formation. Scale bar is equal to 10 μm.

To test whether Abi1 can modulate the activity of Eps8 in cultured cells we examined the effect of co-expressing Eps8 and Abi1 on actin architecture. Similar to data reported previously [9], simultaneous expression of Eps8-myc and Abi1-GFP in B16F1, MDA-MB231, and MDA-MB231BO cells resulted in remodeling of the actin cytoskeleton characterized by formation of cable-like actin bundles within the cytoplasm (Figure 2A–I). The actin cables were typically found at the ventral surface of the cell and displayed few branches. Eps8 and Abi1 co-localized along the length of the actin cables. Interestingly, Abi1 was not enriched with Eps8 in filopodia-like structures (Figure 2, arrowheads in A-C), suggesting that Abi1 may not contribute to Eps8-function at the plasma membrane. More than 95% of transfected cells displayed the actin phenotype shown. Expression of Abi1 alone (data not shown) or an Abi1 mutant (Abi1DY) unable to bind Eps8 [22] failed to stimulate actin cable formation (Figure 2J–L), indicating that the ability of Eps8 to induce actin cables is dependent on its interaction with Abi1.

### Eps8 induces actin remodeling in Xenopus embryos

**Figure 3:**
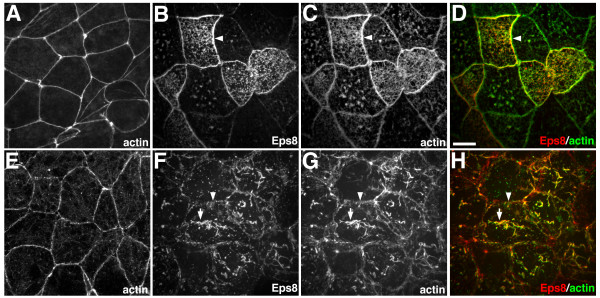
**Eps8-induced actin remodeling in *Xenopus *embryos**. Distribution of actin (A,C,D,E,G,H) revealed by phalloidin staining and Eps8-myc (B,D,F,H) revealed by 9e10 anti-*c-myc *antibody in animal cap cells of *Xenopus *embryos. In control animal caps, actin filaments are enriched at cell-cell junctions in superficial epithelial cells (A) and the cortex of deep cells (E). (B-D) In superficial epithelial cells, Eps8 expression causes an enrichment of actin filaments at cell-cell junctions (arrowheads). (F-H) In deep cells facing the blastocoel, Eps8 expression induces the formation of actin cables (arrowheads). (D,H) Eps8 is red and actin is green in merged images. Scale bar is equal to 10 μm.

To further examine the role of Eps8 in regulating actin architecture, we utilized *Xenopus *animal cap explants, which provide a powerful system for analyzing protein localization and function in vivo. Animal caps explants are dissected from blastula stage embryos and consist of an outer polarized epithelium and 2–3 layers of non-epithelial deep cells. We found that expression of Eps8 has different effects on actin organization in superficial epithelial cells versus deep cells. In control explants, actin filaments are enriched at apical cell-cell junctions in superficial epithelial cells (Figure 3A) and at the cortex of deep cells facing the blastocoel (Figure 3E). In superficial epithelial cells, Eps8 expression caused an accumulation of actin filaments at sites of cell-cell contact in apparent association with adherens junctions (Figure 3B–D, arrowheads). In contrast, Eps8 expression induced the formation of cable-like actin structures within the cytoplasm of deep cells (Figure 3F–H, arrows) and modified the morphology of actin filaments at the cell cortex (Figure 3F–H, arrowheads). The morphology and length of the actin structures in deep cells was variable; long, unbranched filaments were observed in cortical regions in association with the free membrane domain that faces the blastocoel, whereas thick actin bundles were often seen throughout the cytoplasm. Staining of animal caps with anti-*myc *antibodies showed that Eps8-myc localized along the length of actin filaments in both deep and superficial cells (Figure 3D,H; co-localization appears yellow). Thus, Eps8 associates with actin filaments and can dramatically affect the organization of actin in *Xenopus *animal cap cells as it does in cultured cells.

### Abi1 modulates the activity of Eps8 in Xenopus embryos

**Figure 4:**
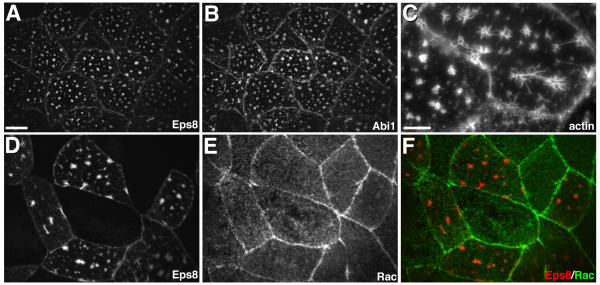
**Abi1 modulates Eps8-induced actin remodeling in a Rac-independent manner**. Distribution of Eps8-myc (A,D), Abi1GFP (B), actin (C) revealed by phalloidin staining, and Rac (E,F) in *Xenopus *animal cap cells. (A-C) Simultaneous expression of Eps8 and Abi1 induce the formation of actin stars at the apical surface of superficial epithelial cells. (D-F) Endogenous Rac is not recruited to Eps8/Abi1-induced actin stars. (F) Eps8 is red and Rac is green in the merged image. Scale bar is equal to 10 μm in A,B and D-F and to 5 μm in C.

To test whether Abi1 can regulate Eps8 function in *Xenopus *embryos, we co-expressed Eps8-myc and Abi1-GFP in animal cap cells and analyzed the localization of Eps8, Abi1, and actin by confocal microscopy. We found that when expressed alone, Abi1-GFP localized to small aggregates found throughout the cytoplasm and did not affect actin organization (data not shown). In contrast, simultaneous expression of Eps8-myc and Abi1-GFP induced the formation of star-like actin structures in superficial epithelial cells of the animal cap (Figure 4A–C). Actin stars were found at the apical surface and consisted of actin-containing spikes radiating from a central actin foci or short bundle. The actin stars did not appear to protrude from the apical surface and Eps8 and Abi1 co-localized with actin in the stars. Since Eps8 and Abi1 facilitate signaling through Rac in cultured cells we tested whether actin star formation was dependent on Rac. In control animal caps, endogenous Rac was enriched at the cell cortex in association with cell-cell junctions (data not shown). In animal caps expressing Eps8 and Abi1, Rac was not recruited to the actin stars (Figure 4D–F) suggesting that Rac activity is not required for actin star formation. In agreement with this idea, expression of dominant negative Rac (RacN17) failed to inhibit Eps8/Abi1-induced actin star formation (data not shown). Thus, Abi1 modulates Eps8 activity in *Xenopus *and Eps8 and Abi1 can stimulate actin remodeling in a Rac-independent manner.

### Recruitment of Actin Regulatory Proteins to Eps8/Abi1-induced actin structures in Xenopus

**Figure 5:**
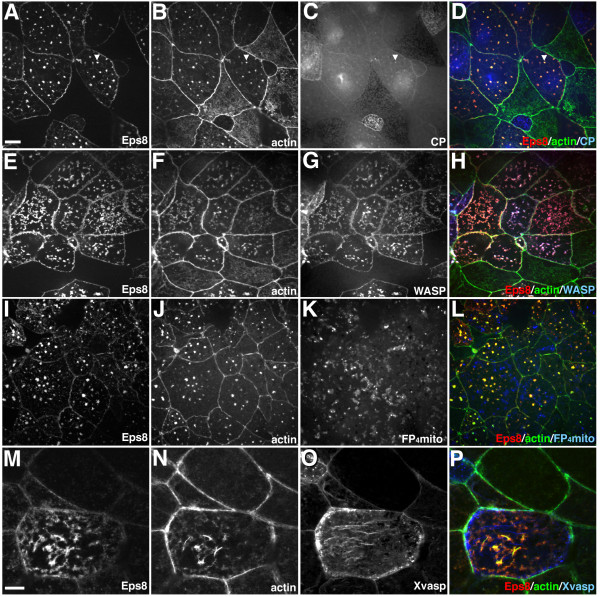
**Regulation of Eps8/Abi1-induced actin remodeling in *Xenopus***. Distribution of Eps8-myc (A,E,I,M; red in D,H,L,P), actin (B,F,J,N; green in D,H,L,P), CP-GFP (C, blue in D), N-WASP-GFP (G, blue in H), FP_4_-mito-GFP (K, blue in L), and Xvasp-GFP (O, blue in P). (A-D) CP does not block formation of actin stars. (E-H) N-WASP is recruited to Eps8/Abi1-induced actin stars. Actin star formation is not altered in response to inhibition of Ena/VASP activity (I-L) or increased levels of Xvasp (M-P). Scale bar is equal to 10 μm in A-L and 5 μm in M-P.

Eps8 has been shown to possess Abi1-dependent barbed-end capping activity in vitro [9], suggesting that the effects we observed in *Xenopus *may be due to increased capping of actin filaments. To test this idea, we analyzed whether expression of capping protein induced similar changes in actin organization. Capping protein (CP) is an α/β heterodimer that is thought to provide the major barbed-end capping activity in eukaryotic cells [23,24]. In these experiments, animal caps expressing both the α and β subunits of CP were examined for changes in actin filament distribution. In addition, since both the α and β subunits were GFP-tagged, their expression was confirmed by Western blot analysis using anti-GFP antibodies (data not shown). We found that expression of CP had no effect on actin organization in animal cap cells (data not shown). In addition, we found that expression of capping protein did not block the formation of Eps8/Abi1-induced actin stars, although low levels of capping protein were found to co-localize with the actin stars (Figure 5A–D, arrowhead). Thus, the formation of actin stars does not directly correlate with enhanced capping protein activity nor does enhanced capping protein activity affect Eps8/Abi1-induced remodeling of the actin cytoskeleton.

WASP/Scar proteins play an important role in stimulating actin filament nucleation by the Arp2/3 complex [25-27]. To test whether the formation of actin stars involves recruitment of WASP proteins we analyzed the distribution of N-WASP-GFP in animal cap cells expressing Eps8 and Abi1. N-WASP co-localized with Eps8 and actin (Figure 5E–H), indicating that WASP proteins are recruited to Eps8/Abi1-induced actin structures. We also tested whether N-WASP activity is required for Eps8/Abi1-induced actin star formation by co-expressing Eps8, Abi1 and a dominant negative form of N-WASP (N-WASP-CA). We found that N-WASP-CA expression did not significantly alter the actin structures induced by Eps8 and Abi1 (data not shown). These data suggest that Eps8 and Abi1 can recruit actin nucleators to specific sites in the cell, although N-WASP function may not be strictly required for Eps8/Abi1-induced actin remodeling.

Members of the Ena/VASP family are critical regulators of actin filament dynamics and are thought to antagonize actin filament capping at the leading edge of migrating cells [28]. Given this central role, we tested whether increased or decreased Ena/VASP activity would affect Eps8/Abi1-induced actin star formation. Expression of a dominant negative protein (FP_4_-mito-GFP, [28,29]) that specifically neutralizes the function of all Ena/VASP proteins was used to knockdown Ena/VASP activity whereas expression GFP-tagged *Xenopus *VASP (Xvasp) was used to increase Ena/VASP activity. The ability of the FP_4_-mito dominant negative to mis-localize Ena/VASP proteins in *Xenopus *was confirmed by showing that it caused the redistribution of endogenous Ena from the cell periphery to the mitochondria surface (data not shown). We found that neither FP_4_-mito-GFP (Figure 5I–L) nor Xvasp-GFP (Figure 5M–P) had an effect on the presence of Eps8/Abi1-induced actin stars. In addition, Xvasp-GFP did not co-localize with the actin stars, indicating that Ena/VASP proteins are not recruited to these actin structures (Figure 5M–P).

### Eps8 recruits Dsh to the membrane and actin filaments

**Figure 6:**
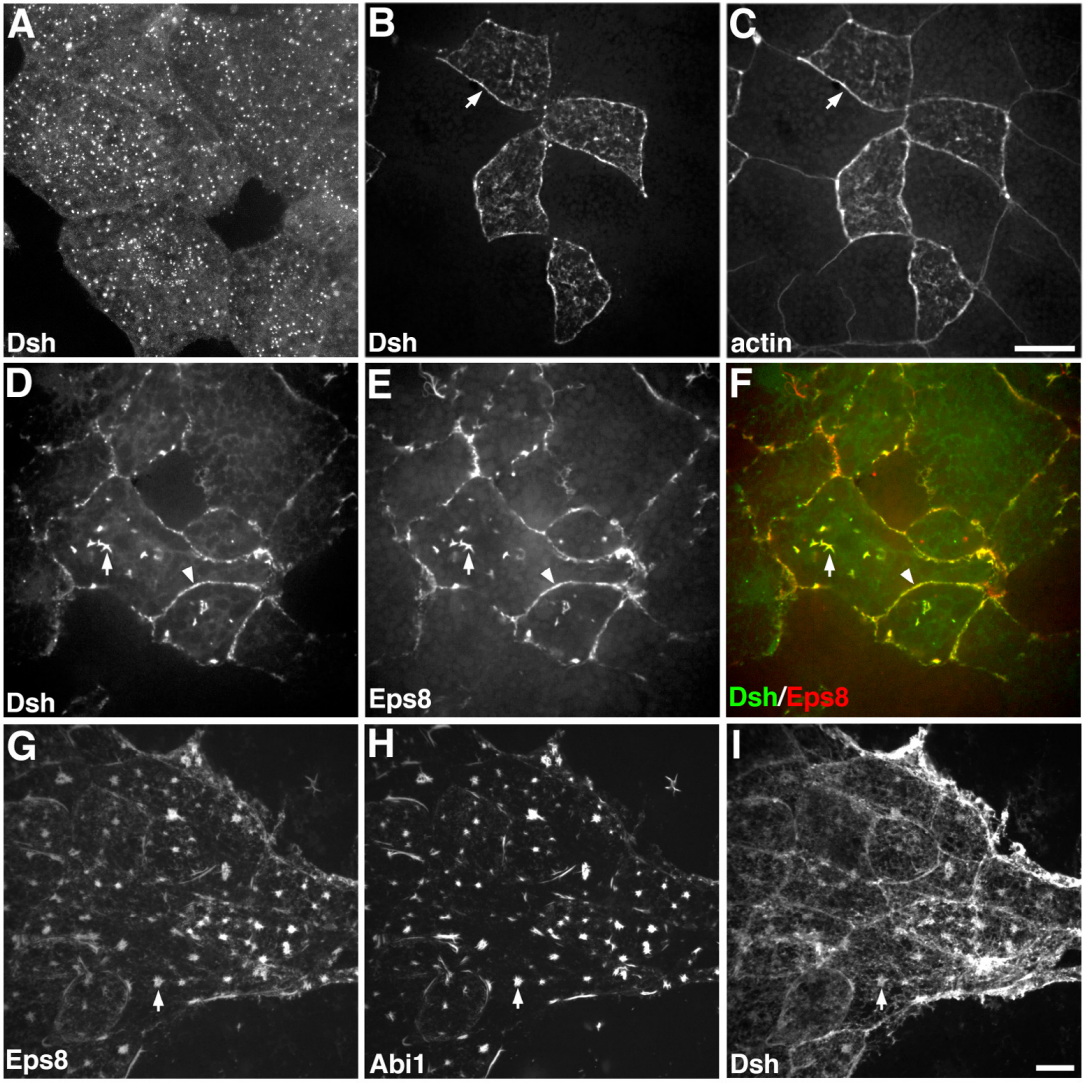
**Dsh is recruited to the plasma membrane and actin filaments in response to Eps8 expression**. Distribution of Dsh-GFP (A,B,D, green in F), actin (C), Eps8-myc (E,G, red in F), Abi1-GFP (H), and Dsh-flag (I) in *Xenopus *animal cap cells. (A) Localization of Dsh-GFP in control animal caps. (B,C) In response to Eps8 expression, Dsh is recruited to the membrane and cell-cell junctions in superficial epithelial cells where it co-localizes with actin (arrows). (D-F) In deep cells facing the blastocoel, Eps8 induced the recruitment of Dsh to cytoplasmic actin cables and the cell cortex where Dsh co-localized with Eps8 (co-localization appears yellow). (G-I) Co-localization of Eps8, Abi1, and Dsh in animal caps cells (arrows mark site of co-localization). Scale bars are equal to 10 μm.

Previous studies have reported that Eps8 can bind the Wnt signaling protein Dsh [11], which is required for the transduction of both canonical and non-canonical Wnt signals [13]. Since Dsh is required for cell polarization and convergent extension movements during gastrulation [14-16,30,31], we hypothesized that the formation of an Eps8/Dsh complex may be important for regulating Dsh localization and function during gastrulation. To test this idea, we asked whether Eps8 interacts with Dsh in animal cap cells. When expressed alone, Dsh-GFP displays a punctate cytoplasmic distribution in animal cap explants (Figure 6A) [32]. Expression of Eps8 caused a dramatic redistribution of Dsh-GFP to the plasma membrane and cytoplasmic actin filaments where it co-localized with actin and Eps8 (Figure 6B–F). In superficial epithelial cells, Dsh was recruited to cell-cell junctions (Figure 6B,C; arrow) and in deep cells Dsh was recruited to cytoplasmic actin cables (Figure 6D–F; arrow) and the cell cortex (Figure 6D–F; arrowhead). Furthermore, we found that epitope-tagged forms of Dsh, Eps8, and Abi1 co-localize in animal cap cells (Figure 6G–I) suggesting that they can form a tri-complex in vivo. These data provide evidence that Eps8 interacts with and may regulate the distribution and/or function of Dsh through recruitment of Dsh to the membrane and actin filaments.

### Identification and developmental expression of Xenopus Eps8

**Figure 7:**
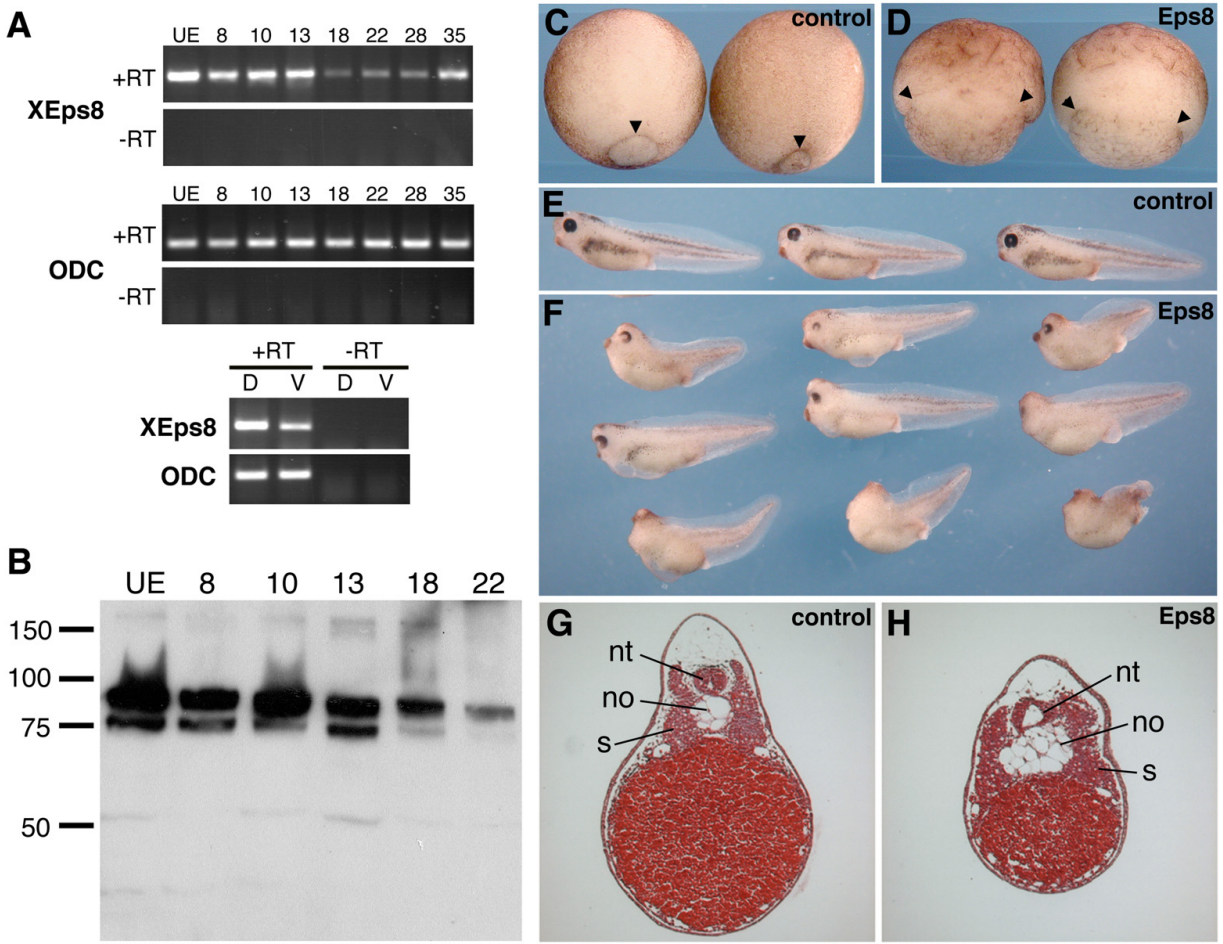
**Mis-expression of Eps8 results in gastrulation defects**. (A) RT-PCR analysis of the developmental expression of *XEps8*. ODC serves as a control for RNA isolation and reverse transcription. (B) Western blot probed with anti-XEps8 antibodies show that XEps8 protein is provided maternally and is present in gastrula, neurula, and tailbud stage embryos. (C) Control and (D) Eps8-injected embryos at stage 12. In control embryos the blastopore is well formed and has progressed vegetally (arrowheads). In contrast, Eps8-injected embryos display severe buckling of tissue above the blastopore (arrowheads) and a disorganized blastopore lip that is delayed and malformed. (E) Control stage 37/38 embryos. (F) Eps8-injected embryos show a range of phenotypes including microcephaly, cyclopia, and shortening and arching of the A-P axis. Top = low dose (50 pg); middle = intermediate dose (200 pg); bottom = high dose (1 ng). (G,H) Histological analysis of Eps8-injected embryos shows that Eps8 expression causes a broadening of the notochord (no) and disorganization of the neural tube (nt) and somites (so).

Eps8 can interact with Dsh and is thought to play an important role in regulating actin remodeling in motile cells, raising the possibility that Eps8 might be a key regulator of cell movements during gastrulation in vertebrate embryos. To begin to address the role of Eps8 during embryonic development, we performed in silico analyses to identify the *Xenopus *ortholog of *Eps8*. Searches of the TIGR (TC263683) and NCBI (MGC81285; Image 6631907) databases led to the identification of cDNAs that encode *Xenopus Eps8 *(*XEps8*). The predicted XEps8 protein shows a high degree of sequence identity with both mouse and human Eps8 and contains the conserved PTB, SH3, and C-terminal effector domains. The developmental expression of *XEps8 *transcripts was determined by RT-PCR. We found that *XEps8 *transcripts are provided maternally and are present throughout development (Figure 7A). We also found that *XEps8 *is expressed in isolated dorsal and ventral marginal zone tissue of gastrula stage embryos and that levels of *XEps8 *are higher in dorsal marginal regions compared to ventral regions (Figure 7A). Finally, we probed blots of embryonic lysates with anti-XEps8 polyclonal antibodies and found that XEps8 protein appears as a doublet and is present in unfertilized eggs, gastrula, and neurula stage embryos (Figure 7B). These analyses show that *XEps8 *is expressed at the relevant time and place to regulate cell movements during gastrulation.

To test the requirement for *XEps8 *during development, we utilized a morpholino (MO) antisense oligonucleotide targeted to the 5'-untranslated region to specifically knockdown levels of XEps8 protein during development. We found that the XEps8 MO could specifically block the expression of a myc-tagged version of XEps8, but injection of the XEps8 MO into 4-cell stage embryos resulted in embryos with no apparent phenotype (data not shown). The lack of a knockdown phenotype is not surprising since *Eps8*^-/- ^mice also displayed no obvious phenotype [6]. Since Eps8 is a member of a multi-gene family, we searched TIGR and NCBI databases for additional *Xenopus *Eps8 genes and found evidence for a second *XEps8 *gene as well as three *XEps8-like *genes. Therefore, the lack of a phenotype in XEps8 knockdown embryos is likely due to the expression of multiple *XEps8 *family members, including *XEps8L1*, *XEps8L2*, and *XEps8L3*, during early development (Roffers-Agarwal and Miller, unpublished results). Thus, assessing the role of Eps8 proteins in *Xenopus *will require novel knockdown techniques capable of simultaneously and specifically inhibiting the activity of multiple gene products during early development.

### Expression of Eps8 disrupts cell movements during gastrulation

Since knockdown experiments produced negative results, we performed mis-expression experiments to test whether altering Eps8 activity would affect cell movements during gastrulation. Synthetic mRNA encoding mouse Eps8-myc or GFP as a control was injected into the equatorial region of both dorsal blastomeres at the 4-cell stage and resulting embryos were then examined for developmental abnormalities. Defects in Eps8-injected embryos were first apparent at stage 10.5 (early gastrula). At this stage, control embryos formed a well-defined dorsal lip indicative of the onset of gastrulation movements and involution of dorsal mesoderm. In contrast, Eps8-injected embryos showed a delay in the formation of the dorsal lip and when observed, the lip was disorganized (data not shown). By stage 12, Eps8-injected embryos displayed a severe delay in blastopore closure and buckling of tissue above the blastopore (Figure 7D). Eps8-injected embryos eventually complete gastrulation and tadpoles displayed a phenotype including a shortened and arched anterior-posterior axis and head defects (Figure 7F). The defects caused by Eps8 are dose dependent; low doses (50 pg) of Eps8 result in cyclopia and a shortened A-P axis, moderate doses (200 pg) show varying degrees of cyclopia, microcephaly, and shortening and arching of the A-P axis, and high doses (1 ng) result in varying degrees of anencephaly, shortening and arching of the A-P axis, and spina bifida. Control, GFP-injected embryos appeared normal at all stages examined (Figure 7C,E). These data are consistent with the idea that Eps8-induced actin re-organization leads to defects in cell movements during gastrulation in *Xenopus*.

The gross morphological defects caused by dorsal expression of Eps8 could be the result of defects in convergent extension or inhibition of mesoderm development, both of which would give superficially similar phenotypes. In order to distinguish between these two possibilities we performed histological analysis on injected embryos (Figure 7G,H). Histological sections of Eps8-injected embryos demonstrated that notochord, somites, and neural tissue are all present, showing that expression of Eps8 does not globally perturb specification of mesodermal or neural cell fates. Instead, expression of Eps8 resulted in broadening of the notochord along the mediolateral axis and morphological defects in the neural tube and somites. The widening of the notochord is consistent with the idea that expression of Eps8 impairs convergent extension movements of the axial mesoderm.

**Figure 8:**
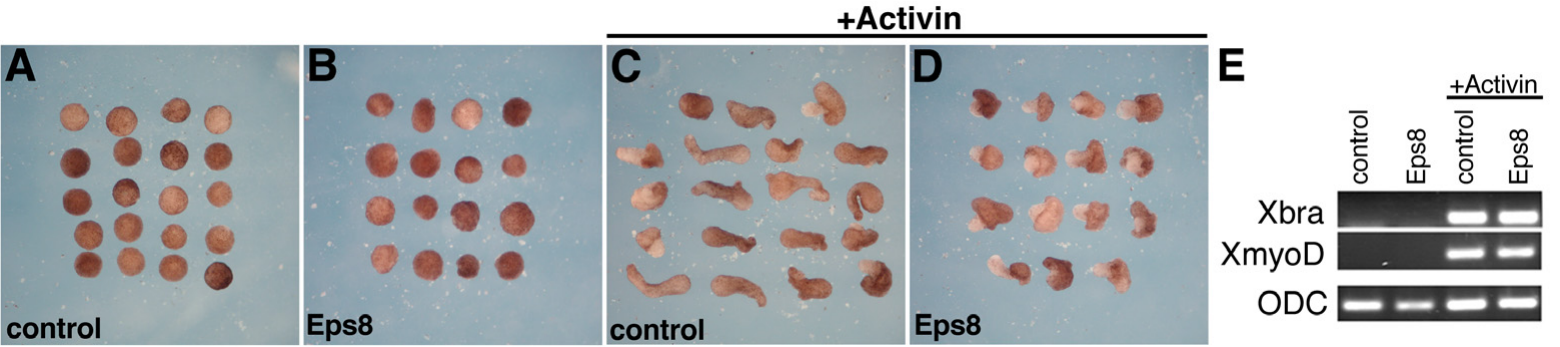
**Eps8 blocks elongation of activin treated animal caps**. (A,B) Control and Eps8-injected animal cap explants remain rounded in the absence of activin. (C,D) Control explants elongate extensively in the presence of activin, whereas Eps8 expression inhibits elongation. (E) RT-PCR analysis shows that Eps8 expression does not block activin-mediated induction of the mesodermal markers Xbra and MyoD. ODC is a control for mRNA isolation, reverse transcription, and gel loading.

Analysis of activin-induced elongation of animal cap explants provides a powerful assay for studying the cell movements associated with gastrulation [14,33,34]. In these experiments, the animal pole region of an embryo is removed at the blastula stage and placed in culture. Untreated animal cap explants and caps expressing Eps8 differentiate into atypical epidermis and remain rounded (Figure 8A,B) whereas addition of recombinant activin induces mesodermal differentiation, convergent extension movements, and elongation of uninjected explants (Figure 8C). We found that expression of Eps8 inhibits activin-induced elongation of animal cap explants (Figure 8D). The failure of activin-induced animal caps to elongate was not caused by a block in mesoderm induction since both Xbra (pan-mesoderm) and XmyoD (paraxial mesoderm) were expressed in control and Eps8-injected animal caps following activin treatment (Figure 8E).

## Discussion

Here, we have investigated how Eps8 regulates actin filament architecture and how this activity impacts cell movements during gastrulation. Our results, together with previous studies, provide evidence that Eps8 plays multiple roles in regulating the actin cytoskeleton and that these functions are influenced by the participation of Eps8 in multi-protein actin regulatory complexes.

Based on in vitro studies, Eps8 is hypothesized to promote capping of actin barbed-ends in an Abi1-dependent manner [9]. Our findings suggest that in addition to its proposed role as a barbed end capping protein, Eps8 might play additional roles in regulating actin organization in vivo. This idea is supported by the observation that Eps8 expression resulted in enhanced formation of actin-rich filopodia-like structures in cultured cells and enhanced formation of actin bundles and accumulation of actin at cell-cell junctions in *Xenopus *embryos. The presence of the filopodia-like structures on the dorsal surface of cells suggests that they are protrusive in nature and do not represent retraction structures, which are typically associated with sites of cell adhesion. Additional studies examining the dynamics of these Eps8-induced structures will help clarify the origin and nature of these structures. In addition, we found that Abi1 modulated Eps8 activity, promoting the formation of actin cables in cultured cells and actin stars in *Xenopus*, suggesting that Eps8 can regulate actin dynamics through Abi1-dependent and -independent mechanisms. Consistent with this idea, Abi1 did not co-localize with Eps8 at the tips of the filopodia-like structures in cultured cells suggesting that additional regulators of Eps8 remain to be identified.

The correlation between Eps8 expression and enhanced formation of filopodia-like structures and actin cables is consistent with the idea that Eps8 may regulate actin filament elongation in vivo. Regulation of barbed-end elongation and filopodia formation is thought to involve a balance between barbed-end capping and anti-capping activities. Proteins such as CP are hypothesized to block elongation and favor formation of a dendritic network [35], whereas proteins including Ena/VASP proteins, which antagonize capping, are hypothesized to promote actin filament elongation and filopodia formation [28,36,37]. Our work examining the regulation of Eps8 activity by CP, N-WASP, and Ena/VASP in *Xenopus *yielded largely negative results, however, making it difficult to discern the relative contribution of Eps8 capping activity versus other potential modes of activity in the regulation of actin architecture. Further biochemical analyses will help elucidate the molecular mechanism(s) by which Eps8 regulates actin dynamics in vivo.

Previous work [6,7,9,38] and our results show that the ability of Eps8 to modulate actin organization is regulated by its interaction with distinct binding partners such as Abi1. We found that Abi1 can modulate Eps8 activity in cultured cells and *Xenopus *embryos. Abi1 binds to the SH3 domain of Eps8 [38,39] and it has been proposed that this binding may alter the conformation or activity of the adjacent actin-binding domain of Eps8 [9]. The mechanism by which Abi1 might regulate Eps8 activity remains unclear, but may involve recruitment of additional regulatory factors such as Dsh, Sos1, and Rac to the Eps8/Abi1 complex [7,38]. In addition, our work shows that N-WASP is recruited to Eps8/Abi1-induced actin stars suggesting that the Eps8/Abi1 complex interacts either directly or indirectly with actin nucleating factors. This idea is supported by the observation that Eps8 can facilitate actin-based motility of N-WASP-coated beads in vitro in the presence of Arp2/3, ADF/cofilin, and profilin [9]. Further studies will be required to examine how Abi1 modulates Eps8 activity and how Eps8 works with Abi1 and other regulatory factors to control actin organization in vivo.

Eps8 has been shown to bind Dsh [11], a component of the Wnt signaling pathway that is required for transduction of canonical Wnt/β-catenin and non-canonical signals [12,13]. Here, we have shown that Eps8 expression recruits Dsh to actin filaments and the cell membrane in *Xenopus*. These data are significant because the role of Dsh in non-canonical Wnt/Polarity signaling is thought to be dependent on its localization to the membrane and its ability to affect cell polarity and migration through regulation of the actin cytoskeleton [14-18]. Dsh activity during gastrulation is dependent on both RhoA and Rac, and the formin homology protein DAAM1 is required for Dsh-mediated activation of RhoA [17,18]. However, a link between Dsh and Rac has not been identified. The Eps8/Abi1/Sos1 complex is required for growth factor stimulated activation of Rac [6], suggesting that Eps8 might provide an important link between Dsh, Rac, and the actin cytoskeleton during development. Consistent with this idea, expression of Eps8 impaired cell movements during gastrulation and Eps8, Abi1, and Dsh co-localize in *Xenopus *suggesting that these proteins can form a tri-complex in vivo. Interestingly, we did not observe an effect of Eps8 on Dsh-mediated induction of Wnt/β-catenin target genes (*siamois *and *Xnr3*, JRA and JRM unpublished results) indicating that Eps8 does not participate in canonical Wnt/β-catenin signaling. Unfortunately, our attempts to analyze the requirement for Eps8 in *Xenopus *were unsuccessful due to the expression of multiple Eps8 family members during early development. Thus, additional studies are necessary to determine the potential role of Eps8 in the transduction of non-canonical Wnt signals and the potential role of Eps8 family members during gastrulation in vertebrates.

## Conclusion

How might Eps8 regulate the actin cytoskeleton in vivo? Our findings together with data from previous studies support the idea that Eps8 might regulate actin architecture in multiple ways. Eps8 can bind to both barbed ends and the sides of actin filaments [9,10] and it is possible that these different modes of actin binding mediate distinct effects on actin architecture in cells. Barbed-end capping activity might regulate actin filament dynamics and stabilize existing filaments whereas an alternative activity might promote the formation and maintenance of actin arrays required for protrusive force generation and cellular structures such as microvilli and filopodia. This idea is consistent with our observation that Eps8 is enriched at the tips of filopodia-like structures and localizes along the length of the filopodia-like structures and actin cables. This model is also in agreement with the observation that Eps8 localizes to microvilli in the intestinal epithelium of *C. elegans *and knockdown of Eps8 is associated with defects in microvilli formation [40]. The formation of actin cables in cells expressing Eps8 and Abi1 and actin clusters in *Xenopus *embryos suggests that Abi1 is a critical modulator of Eps8's activity as an actin regulatory protein. The finding that Eps8 expression impairs cell movements during gastrulation provides further support for this view and underscores the idea that the proper balance of actin assembly, disassembly, and organization is essential for controlling morphogenetic movements during development. Thus, Eps8 has emerged as a critical regulator of actin filament dynamics and further analysis of Eps8 and its binding partners will help shed light on the mechanisms that mediate actin-based motility in vivo.

## Methods

### Expression constructs, antibodies, and cells

Constructs used were: mouse Eps8-myc pCS2+ (Eps8 cDNA provided by Dr. P.P. DiFiore, European Institute of Oncology, Milan [6]), human Abi1-GFP pCS2+ (Abi1 cDNA provided by Dr. Ann Marie Pendergast, Duke University; [41]), human capping protein α-GFP and β-GFP pCS2+ (capping protein cDNAs provided by Dr. Dorothy Schafer, University of Virginia [42]), FP_4_-mito-GFP pCS2+ (FP_4_-mito cDNA provided by Dr. Frank Gertler, MIT; [29]), RacN17 pCS2+ (RacN17 cDNA provided by Dr. Jennifer Westendorf, University of Minnesota), *Xenopus *Dsh-GFP pCS2+ [32], and *Xenopus *Dsh-flag pCS2+ [43]. *Xenopus *N-WASP-mRFP pCS2+, *Xenopus *N-WASP-CA-mRFP pCS2+ and *Xenopus *VASP-GFP pCS2+ were constructed by PCR using full length IMAGE cDNAs obtained from ATCC. Details of vector construction are available upon request. Primary antibodies used were: mouse anti-*c-myc *9e10 [44], mouse anti-flag (Sigma), rabbit anti-Eps8 (Santa Cruz Biotechnology), and anti-Rac (Transduction Labs). Anti-XEps8 rabbit polyclonal antibodies were raised against a peptide corresponding to the carboxyl terminal (NH_2_-SDSGVESFDEGNSH-COOH) conjugated to KLH (Sigma Genosys). A cysteine residue was added to the amino terminus of the peptide to facilitate conjugation to KLH. Secondary antibodies used were: Alexa568 goat anti-mouse, Alexa647 goat anti-mouse, and goat anti-rabbit Alexa568 and Alexa647 (all secondary antibodies were from Molecular Probes). Alexa568 phalloidin (Molecular Probes) was used to visualize F-actin. Cells used were B16F1 mouse melanoma cells (ATCC), MDA-MB231, and MDA-MB231BO (provided by Dr. Douglas Yee, University of Minnesota).

### Cell culture, transfections, and imaging

B16F1, MDA-MB231, and MDA-MB231BO cells were grown in DMEM (CellGro) supplemented with 10% FBS (HyClone) at 5% CO_2_. For transfections, cells were plated on acid washed coverslips and transfected with Lipofectamine (Invitrogen). For imaging, cells were washed once with PBS and fixed in 4% formaldehyde in CSK buffer (10 mM Hepes pH 7.5, 150 mM sucrose, mM EGTA, 0.1% Triton X-100) for 15 min. at room temperature. Alternatively, cells were permeabilized with 0.1% Triton X-100 in PEM buffer (10 mM Pipes pH 7.4, 1 mM EDTA, 1 mM MgCl_2_) for 30 seconds and fixed with pre-warmed 4% paraformaldehyde in PEM buffer for 30 min. at 37°C. Fixed cells were then washed three times in PBS + 0.1% Triton X-100 (PBST), and incubated in PBST, 2% BSA, 10% normal goat serum (NGS) to prevent non-specific binding of antibodies. Staining with primary and secondary antibodies was performed in PBST, 2% BSA, 10% NGS for 2 hours at room temperature. Images were collected using a Zeiss spinning disc confocal microscope and digital images were processed using Adobe Photoshop.

### Embryos, microinjections, imaging, RT-PCR, and Western blotting

*Xenopus laevis *eggs were fertilized in vitro and subsequently de-jellied in 2% cysteine (Sigma Chemical). Embryos were reared in 1/3× Marc's Modified Ringer's (MMR). Embryos were staged according to Nieuwkoop and Faber [45]. Animal cap explants were prepared using a Gastromaster microsurgery instrument (Xenotek Engineering) and cultured in 1× Steinberg's in the presence of 10 ng/ml Activin (R&D Systems). For microinjections, embryos were placed in a solution of 4% Ficoll in 1/3× MMR and injected using a Harvard Apparatus microinjector and a Narashige micromanipulator. Injected embryos were reared in 4% Ficoll in 1/3× MMR supplemented with 10 μg/ml Gentamicin (Invitrogen) to stage 9 then washed and reared in 1/3× MMR + Gentamicin. Capped mRNA for injections was synthesized using mMessage Machine (Ambion) and purified using NucAway columns (Ambion).

For imaging, embryos and explants were fixed in 4% formaldehyde in CSK buffer at room temperature for 30 min., washed three times in PBST, and incubated in PBST, 2% BSA, 10% NGS to prevent non-specific binding of antibodies. Staining with primary and secondary antibodies was performed in PBST, 2% BSA for 2 hours at room temperature. Actin was visualized with Alexa568 phalloidin (Molecular Probes). Images were captured with a Zeiss spinning disk confocal microscope and digital images were processed with Adobe Photoshop.

RT-PCR analysis was performed using total RNA isolated from *Xenopus *explants. cDNA was prepared using SuperScript II reverse transcriptase (Invitrogen) and PCR was performed using GoTaq polymerase (Promega). Primers used for RT-PCR: *XEps8*: forward: 5'-attccctgagatgttgctccg-3', reverse: 5'-tagcagcagcgatttgccc-3'; *XmyoD*: forward: 5'-agctccaactgctccgacggcatgaa-3', reverse: 5'-aggagagaatccagttgatggaaca-3'; *Xbra*: forward: 5'-ggatcgttatcacctctg-3', reverse: 5'-gtgtagtctgtagcagca-3'; and *ODC*: forward: 5'-gccattgtgaagactctctccattc-3', reverse: 5'-ttcgggtgattccttgccac-3'.

Protein lysates for Western blots were prepared by homogenizing embryos in ice-cold lysis buffer (20 mM Tris pH 7.5, 150 mM NaCl, 1 mM EDTA, 1 mM EGTA, 0.5% Triton X-100) supplemented with protease inhibitors (1 mM PMSF, 1 mM pepstatin, 10 μg/ml leupeptin, and 10 μg/ml aprotinin). Homogenates were cleared by centrifugation at 14,000 rpm for 10 min. at 4°C. SDS sample buffer was added to the cleared lysate and boiled for 4 min. prior to separation by SDS-PAGE. Approximately one embryo equivalent was loaded per lane on 10% gels (BioRad). Proteins were blotted to PVDF membrane (BioRad), blots were blocked in 5% milk in TBS + 0.1% Tween, and probed with anti-XEps8 antibodies (1:2000) for two hours at room temperature. Visualization was performed using a horseradish peroxidase conjugated anti-rabbit secondary antibody (Jackson ImmunoLabs) and enhanced chemiluminescence (Pierce).

## List of abbreviations used

Eps8: Epidermal Growth Factor substrate 8, Abi1: Abl Interacting Protein 1, CP: capping protein, Dsh: Dishevelled, RT-PCR: Reverse Transcriptase Polymerase Chain Reaction

## Authors' contributions

JRA participated in analysis Eps8 and Abi1 regulation of actin in B16F1 cells, participated in analysis of Eps8, Abi1, and Dsh interactions in *Xenopus*, performed Eps8/Abi1 interactions with capping protein, N-WASP, and Ena/VASP proteins, and performed RT-PCR study of analysis of Eps8 activity in Wnt signaling. JBX carried out histological analysis of Eps8-injected embryos. JRM participated in experiments analyzing Eps8 and Abi1 regulation of actin in B16F1 cells, performed studies in MDA-MB231 cells, conducted studies examining the role of Eps8 in gastrulation, coordinated the study, and drafted the paper. All authors read and approved the final manuscript.
